# El dentobioma y la mínima intervención en odontología

**DOI:** 10.21142/2523-2754-1003-2022-124

**Published:** 2022-09-28

**Authors:** Denisse Aguilar-Gálvez, José Carlos Maguiña-Mercedes

**Affiliations:** 1 División de Odontopediatría, Carrera de Estomatología de la Universidad Científica del Sur. Lima, Perú. daguilar@cientifica.edu.pe Universidad Científica del Sur División de Odontopediatría Carrera de Estomatología Universidad Científica del Sur Lima Peru daguilar@cientifica.edu.pe; 2 NeuroResearcher, CEO Neuron Perú. Lima, Perú. josecarlosamigo@gmail.com NeuroResearcher, CEO Neuron Perú Lima Perú

**Keywords:** dentobioma, dentobiota, microbioma oral, microbiota oral, mínima intervención, dentobiome, dentobiota, oral microbiome, oral microbiota, minimal intervention

## Abstract

Todos los que observamos a diario las cifras escalofriantes de niños con caries dental y, más recientemente, la mayor frecuencia de alteraciones del esmalte, entre ellas la hipomineralización del molar incisivo, nos ponemos a pensar en qué pasa realmente con ese tejido considerado el más fuerte del cuerpo humano, pero que se doblega frente a un ataque ácido producto del metabolismo bacteriano. Entonces, surge la pregunta: ¿será que el diente ya nace con una predisposición a desarrollar ciertos microorganismos? Esta revisión exhaustiva de la literatura, que resume las perspectivas de los autores, tiene como objetivo explorar el conocimiento sobre el bioma y aplicarlo al órgano dental, así como poner a disposición la definición de dentobioma, como término apropiado para la flora dental. A partir de este conocimiento, se busca entender mejor la ejecución de la filosofía de la mínima intervención y el desarrollo de materiales que deben ser biocompatibles con la estructura dental, pero que además deben de impedir la disbiosis y asentar la homeostasis en el diente.

## Abstract (en)

All of us who observe the chilling figures of children with dental caries on a daily basis and more recently the more frequent presence of enamel alterations and among them incisor molar hypomineralization, we begin to think what really happens with that tissue considered the strongest. of the human body; however, it bows down to an acid attack resulting from bacterial metabolism. And the question arises: Could it be that the tooth is already born with a predisposition to develop certain microorganisms? This comprehensive review of the literature, summarizing the authors' perspectives; aims to explore the knowledge about the biome and apply it to the dental organ and make available the definition of dentobiome, as an appropriate term for dental flora. Starting from this knowledge, apply it to better understand today the execution of the philosophy of minimal intervention and the development of materials that must be biocompatible with the dental structure but must also prevent dysbiosis and establish homeostasis in the tooth.

## INTRODUCCIÓN

Todos imaginamos que la formación de los dientes deciduos y permanentes comienza en la sexta semana de vida intrauterina y, lógicamente, tienen un 50% de contenido genético de cada progenitor. A esta transmisión de caracteres los conocemos como genética; sin embargo, todo aquello que afecte a la madre durante este periodo modificará ese porcentaje; en ese momento comenzamos a hablar de epigenética. ¿Qué pasa entonces después del nacimiento, durante esas casi 118 semanas (los famosos primeros 1000 días del bebé)?

Situémonos en donde todo se inicia: la fecundación, proceso mediante el cual el ADN de un X se une al de una Y, y toda esa información comienza a entremezclarse. Acaso este es el momento en el que también se inicia un *nuevo bioma*. Recordemos que un *bioma* (del griego *bios*, ‘vida’) es un conjunto de ecosistemas que cada ser tiene en su organismo y este nuevo ser es susceptible a tener tal o cual bioma ^1^. Sin embargo, será la gestión de vida de esa incubadora humana que es el vientre materno la que va a definir las características no solamente de que la trasmisión genética se mantenga, sino también de que se vea influenciada positivamente por un buen bioma. Desde ese momento, podemos afirmar intrépidamente que la primera disbiosis ocurre en el momento que esa gestación se vea alterada; esto se conoce también como el paradigma del origen del desarrollo de la salud y la enfermedad ^2^^,^^3^. Entonces, ¿cuáles serían los factores que alteran esa gestión, que también podemos llamar salud materna, durante estas 40 semanas ^4^?

- Una enfermedad infecciosa. La más común es la infección urinaria, seguida del citomegalovirus y, más recientemente, la COVID-19.
- Una enfermedad ocasional. Las más comunes son la diabetes mellitus gestacional, la hipertensión arterial y la preeclampsia.
- Un estado emocional de alerta. La ansiedad, que puede llegar incluso al estrés.

Durante la sexta semana se comienzan a formar los dientes y sucede algo muy interesante para la histología en general. Este órgano se comienza a desarrollar de afuera hacia adentro; sí, es el esmalte el que se forma primero ^5^. Este proceso, llamado amelogénesis, comienza en el útero y está influenciado por factores maternos y ambientales ^6^. Los ameloblastos son un grupo vulnerable de células que pueden ser afectadas por factores estresantes internos o externos inespecíficos durante la vida intrauterina ^7^. Esto significa que el estrés prenatal podría afectar la formación del esmalte y producir un esmalte dental hipomineralizado o más susceptible a la caries ^8^^,^^9^. Lo señalado sigue el llamado “modelo del período crítico”, pero también se han propuesto otros modelos que explican la posible asociación entre las complicaciones del embarazo y la caries dental en la epidemiología del curso de la vida. Aunque en diferentes estudios se ha encontrado una relación positiva entre las complicaciones del embarazo y la caries dental, la evidencia sigue sin ser concluyente ^10^.

Todo hasta aquí está bien y es lo que tenemos en la actualidad; sin embargo, la enfermedad de caries dental y la presencia de hipomineralización en algunos dientes deciduos y permanentes es la consecuencia de una *disbiosis* de esta flora propia del diente, que es dinámica y polimicrobiana, y la precursora directa de enfermedades ^11^. Eso es algo que se debe considerar para enfrentar desde otro ángulo ambas entidades clínicas.

## EL DIENTE Y SU PROPIO ECOSISTEMA: DENTOBIOMA

Sabemos ya que los dientes son parte de la boca y, por lo tanto, del sistema digestivo; sin embargo, vamos a poner en consideración, a partir de este momento, que los dientes son sistemas diferenciados, con sus propios microorganismos, a lo que vamos a denominar *dentobioma*. Ya de una manera particular e histológica, cada diente tiene un sistema binario de protección que, en conjunto, se llama complejo dentino-pulpar y tiene una matriz de protección externa que es el esmalte que, sin embargo, es la más susceptible a los cambios del microbiota oral.

De esta manera, podríamos decir que, dependiendo de cada individuo y sus diferentes factores asociados (edad, etnia, costumbres, hábitos, dieta, etc.), cada diente desarrollará también su propia *dentobiota*.

La boca es considerada el segundo laboratorio con la comunidad microbiana más grande y diversa que se encuentra en el cuerpo. La microbiota oral crece como biopelículas de múltiples especies en las superficies expuestas, y se pueden aislar entre 100 y 300 especies de un solo individuo ^12^. El conocimiento del microbioma asociado a la caries es clave para la planificación de enfoques que reviertan la disbiosis. Una evaluación constante sobre la variedad de especies bacterianas sería ideal para establecer evaluaciones y tratamientos según el riesgo. Las diversidad del microbioma estable se comporta y tiene un rol importante en la evolución de las enfermedades ^2^.

El estudio del microbioma sugiere un papel para el ensayo de especies cariogénicas putativas seleccionadas en enfermedades más agresivas. Sin embargo, para muchas poblaciones con progresión de caries, es probable que se justifique ensayos de múltiples especies para determinar el perfil de caries de la población o los individuos bajo estudio. En un estudio que trabajó con una población con una experiencia de caries más baja, se obtuvo una detección de *Streptococcus mutans* más baja o nula, pero albergaba otros taxones acidogénicos en el microbioma ^3^. Estos conocimientos son vitales para poder enfocar la gestión preventiva, pero qué pasa cuando nos involucramos con un mundo aún más exclusivo o específico, porque la boca es el universo compuesto por dientes, encía, mucosa, lengua, entre otros tejidos. El diente, entonces, se convierte en nuestro principal actor o motivo de conversación.

**Figura 1:**
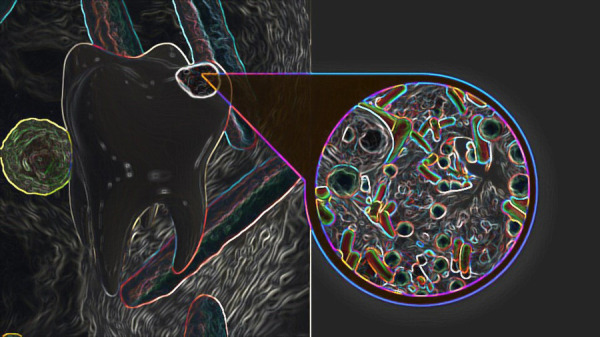
El *dentobioma*

El *dentobioma* es la comunidad de microrganismos adscritos exclusivamente al diente (esmalte, dentina, cemento y pulpa dental); en ese sentido, dependerá de la morfología, la calidad del diente, su composición, sus alteraciones y su tipo (si es deciduo o permanente) el *dentobioma* que va a tener (Figura 1). El dentobioma se analizará mediante la secuenciación de ampliación de ARNr 16S, combinada con el algoritmo de búsqueda basado en BLASTN para la identificación de especies.

Tal vez el estudio, más cercano para acompañar a esta descripción es el de Ribeiro ^13^, quien, luego de recolectar biopelículas de las superficies oclusales de molares deciduas, encontró comunidades altamente polimicrobianas, correspondientes a 10 filos bacterianos, 25 clases, 29 órdenes, 58 familias, 107 géneros y 723 especies. Estamos entonces frente a algo más específico y a partir de este momento, lo vamos a relacionar con la otra alteración que desde hace décadas pudo ser el falso positivo del diagnóstico de caries: la hipomineralización de molares, ya sean deciduos (HMD) o permanentes (HMI).

### Importancia de conocer el dentobioma para aplicar la mínima intervención en odontología

El concepto de la *mínima intervención* va desde la prevención de la instalación de la enfermedad, realizando primero un diagnóstico correcto de riesgo y abordaje de la lesión cariosa, para luego realizar un plan de tratamiento no invasivo; con el fin de paralizar la progresión de la enfermedad y un abordaje restaurador para remover la mínima cantidad de tejido saludable utilizando materiales permanentes ^14^.

La “remoción parcial del tejido cariado” es un abordaje conservador en el que apenas la dentina necrótica desorganizada es removida y, después, la cavidad es rellenada con una restauración final en la misma sesión. ¿Qué pasa entonces con las bacterias que se quedan al no retirar todo el tejido infectado? La presencia de bacterias por sí solas no determina la progresión de la lesión cariosa, depende de la actividad metabólica en la superficie dental. Además, se ha demostrado que las lesiones no cavitadas (activas o inactivas) también tienen presencia de microrganismos ^15^.

Comenzamos a partir de este primer estudio de revisión a desarrollar y explicar cómo el dentobioma y la dentobiota tienen que ver con las lesiones cariosas cavitadas y las que se extienden a la dentina que han sido tratadas mediante la eliminación selectiva del tejido cariado; la eliminación gradual del tejido cariado, el sellado de las lesiones cariosas con materiales selladores, el sellado con materiales preformados coronas de metal (técnica de Hall, HT) y el control de cavidades no restaurativas (NRCC).
